# High fiber diet attenuate the inflammation and adverse remodeling of myocardial infarction *via* modulation of gut microbiota and metabolites

**DOI:** 10.3389/fmicb.2022.1046912

**Published:** 2022-12-21

**Authors:** Jinxuan Zhao, Wei Cheng, He Lu, Anqi Shan, Qi Zhang, Xuan Sun, Lina Kang, Jun Xie, Biao Xu

**Affiliations:** ^1^Department of Cardiology, State Key Laboratory of Pharmaceutical Biotechnology, The Affiliated Hospital of Nanjing University Medical School, Nanjing Drum Tower Hospital, Nanjing University, Nanjing, China; ^2^Department of General Surgery, Jiangsu Province Hospital of Chinese Medicine, Affiliated Hospital of Nanjing University of Chinese Medicine, Nanjing, China; ^3^Nanjing Drum Tower Hospital, Clinical College of Nanjing University of Chinese Medicine, Nanjing, China; ^4^Department of Rehabilitation, Nanjing Drum Tower Hospital, The Affiliated Hospital of Nanjing University Medical School, Nanjing University, Nanjing, China

**Keywords:** myocardial infarction, high fiber diet, gut microbiota, metabolomics, inflammation, fibrosis

## Abstract

**Introduction:**

High intake of dietary fiber is associated with lower incidence of cardiovascular diseases. Dietary fiber, functions as a prebiotic, has a significant impact on intestinal bacteria composition and diversity. The intestinal flora and metabolites generated by fermentation of dietary fiber not only affect the health of intestine but also play a role in many extra-intestinal diseases, such as obesity, diabetes and atherosclerosis. However, the role and the mechanism by which a high fiber diet contributes to the development of myocardial infarction is still unclear.

**Methods and results:**

Here we used an *in vivo* mouse model to investigate whether dietary fiber intake could protect against myocardial infarction. Our study demonstrated high fiber diet significantly improved cardiac function, reduced infarct size and prevented adverse remodeling following myocardial infarction. The protective effects of high fiber diet had a strong relation with its attenuation of inflammation. Moreover, we observed that high fiber diet could modulate the composition of intestinal flora and differentially impacted metabolites production, including the biosynthesis of bile acids and linoleic acid metabolism.

**Conclusion:**

Overall, the findings of this study provided mechanistic insights into the curative effect of dietary fiber on myocardial infarction with a specific emphasis on the potential role of microbiota-metabolism-immunity interactions.

## Introduction

Myocardial infarction (MI) remains one of the leading causes of disability and death worldwide, whose continuously increasing prevalence is associated with unhealthy diet and sedentary lifestyles (Livingstone et al., 2021). A growing number of clinical trials have declared that an abundant dietary intake of vegetables and fruit is accompanied by a lower incidence of atherosclerosis, MI and reduced blood pressure (Wang et al., 2014; Gao et al., 2021). However, the role and the mechanism by which a high fiber diet contributes to the development of MI itself is uncertain. Attention has been focused on the commensal bacteria living in the gastrointestinal tract that relies upon dietary fiber as an energy source (Rowland et al., 2018; Zhang et al., 2020).

Gut microbiota, the most abundant symbiotic compartment in the body, is essential for human health. Disturbance of gut microbial community, namely dysbiosis, may promote the development of metabolic malfunction, including obesity (Gomes et al., 2018), diabetes (Gurung et al., 2020), hypertension and atherosclerosis (Verhaar et al., 2020), thus increasing the risk of MI. Recently, emerging evidence indicated that intestinal flora dysbiosis not only increased the susceptibility to MI but also affected cardiac repair post MI (Liu et al., 2017; Zhou et al., 2020; Han et al., 2021). Gan et al. (2014) reported that probiotic supplementation could significantly improve cardiac function and prevent adverse remodeling in an experimental MI model. Dietary fibers have been found to induce a more diversified gut microbiota that produces metabolites capable of modulating local and systemic inflammation and maintaining host immune homeostasis in multiple ways (Cronin et al., 2021; Wastyk et al., 2021). Growing evidence declares that the inflammatory response occurring following MI plays a pivotal role in the development of heart failure *via* exacerbating tissue damage and promoting the myocardial fibrosis (Prabhu and Frangogiannis, 2016). Given the immunomodulatory effects of gut microbiota and its metabolites (Rooks and Garrett, 2016), we therefore hypothesized that dietary fibers induced gut microbiota changes might have an impact on the efficiency of repair after MI. In this study, we investigated the effect of high fiber diet on cardiac repair following MI and the impact of intestinal microbiota-metabolite interaction induced by dietary fibers in heart-gut axis.

## Materials and methods

### Animal and induction of myocardial infarction

All procedures with animals were performed in accordance with the “Animal research: reporting of *in vivo* experiments” (ARRIVE) guidelines and approved by the Institutional Ethics Committee of Nanjing Drum Tower Hospital (Approval No. 20011141). Male C57BL/6 mice were purchased (8 weeks old) from the Model Animal Research Center of Nanjing University and housed in a temperature (22 ± 1°) and humidity (65–70%) controlled room, with a 12-h light–dark cycle.

Diet interventions used in this study were control diet (normal chow, 47.6% fiber) and high fiber diet (72.7% fiber, SF11-025, Specialty Feeds) as previously described (Marques et al., 2017). To induce MI in mice model, male C57Bl/6 mice were anesthetized with 1.5–2% isoflurane and artificially ventilated using a rodent ventilator. After ventilation, thoracotomy was performed at the fourth intercostal space to expose the heart and left anterior descending coronary artery (LAD). LAD was permanently ligated at 2–3 mm distal to the left atrial appendage as previously described (Li et al., 2019). Sham-operated animals underwent the same surgical procedures without ligation of the exposed LAD. Male C57Bl/6 mice were randomly assigned to various groups: sham control (sham-operated, control diet), sham fiber (sham-operated, high fiber diet), MI control (MI-operated, control diet), MI fiber (MI-operated, high fiber diet). Mice received the control diet or high fiber diet from 3 weeks prior to operation to the end of the study.

### Echocardiography

Cardiac function was assessed *in vivo* using transthoracic echocardiography (Vevo®2,100 Imaging System, Visualsonics) by experienced users who were blinded to grouping information. Hearts were imaged in 2-D and M-mode and two-dimensional short and long axis were visualized. Dimensions of the left ventricle (LV) diameter were measured from at least three consecutive cardiac cycles and analyzed using the Vevo®2,100 based measurements software.

### Tissue collection

At euthanasia, hearts were arrested in diastole after intraventricular injection of 10% potassium chloride (KCl) and then immediately removed. Cardiac weight index (CWI, mg/g) was determined as the total heart weight (mg) relative to total body weight (g) of the animal. Intestinal tissues, colonic contents and blood were also collected for further analysis.

### Hematoxylin and eosin staining

Four week following MI, hearts were harvested, fixed with 4% phosphate-buffered formalin (pH 7.4), embedded in paraffin, cut into transverse sections (5 μM thickness) and then stained with hematoxylin and eosin (H&E) according to the manufacturer’s protocol. Infarct size measurements were obtained at the base, midpapillary, and apical regions of the hearts stained with H&E. The infarct size was determined as the average ratio of the infarct area to the LV area by using Imaging Pro software.

### Wheat germ agglutinin staining

To determine cardiomyocyte size, we used wheat germ agglutinin (WGA) coupled with Alexa Fluor 488 conjugate (Invitrogen Life Technologies) stain in combination with DAPI according to the protocol described in our previous study (Li et al., 2019; Zhao et al., 2019). Images were acquired using a Lecia fluorescence microscope (Leica, Germany). Only cardiomyocytes with centrally located nuclei were calculated for cell size determination.

### Masson’s trichrome staining

Hearts were embedded in paraffin, cut into 5 μM thick sections, and then stained with Masson trichrome according to the manufacturer’s instruction. To assess interstitial fibrosis level, we randomly selected 10 fields (400× magnification) within the peri-infarct zone and calculated the collagen volume fraction as the blue-stained (collagen) area relative to the total tissue area. The collagen-rich zone around the vessels and the scar were excluded from the analysis.

### Enzyme-linked immunosorbent assay

Blood samples were collected and then centrifuged to isolate serum for inflammatory cytokines measurements. The protein levels of IL-1β, IL-6, and TNF-α were measured using the corresponding mouse enzyme-linked immunosorbent assay (ELISA) kit according to the manufacturer’s protocols (MultiScience, Hangzhou, China).

### Flow cytometry analysis

Peripheral blood samples were collected into heparinized tubes. The left ventricle tissues of peri-infarct area were collected in cold staining buffer (2% FBS, 0.05% NaN_3_ in PBS). For tissue cytometry analysis, single-cell suspensions of tissues were obtained using gentleMACS™ Dissociator (Miltenyi Biotec). Samples were stained with fluorochrome-conjugated antibodies against extracellular or intracellular markers according to the manufacturer’s protocols. The following antibodies were used for extracellular staining: CD11b-FITC (BD Bioscience), F4/80-PerCP/Cy5.5 (eBioscience) and Ly6C-APC (eBioscience). The intracellular fixation and permeabilization kit (eBioscience) was used according to the manufacturer’s protocol for intracellular staining. Cells were then washed with staining buffer and stained with CD206-APC (eBioscience) and iNOS-PE (eBioscience). After incubation with antibodies, blood samples were treated with red blood cell lysis buffer to remove red blood cells and heart tissue samples were filtered through a 70-μM filter. Flow cytometry was performed using FACS Aria flow cytometer (BD Bioscience), and flow cytometry data were analyzed using FlowJo software (TreeStar, Ashland, OR).

### Bacterial DNA extraction and 16S ribosomal RNA gene sequencing

Colonic contents were collected sterilely from the descending colon during euthanasia, flash frozen in liquid nitrogen, and stored at −80°C until further use. Bacterial DNA from colonic contents was extracted using the QIAamp DNA Stool Mini Kit (Qiagen, Hilden, Germany) following manufacturer’s protocol and amplified using primers targeting the V4 region of the 16S rRNA gene using the 515F (5′- GTGCCAGCMGCCGCGGTAA −3′) and 806R (5’-GGACTACHVGGGTWTCTAAT-3′) primers with overhang adapters. After polymerase chain reaction (PCR), barcoded 16S amplicons were purified, quantified and then sequenced on the Illumina HiSeq 2,500 platform. Raw data were filtered and primers were trimmed. The trimmed sequences were then clustered into operational taxonomical units (OTUs) with a threshold of 97% by the UPARSE algorithm. Microbiota bacterial composition analysis was done in QIIME (version 2.0) according to previously published work (Hu et al., 2021; Khan et al., 2022). The sequencing data for our study are available in the NCBI Sequence Read Archive under accession no. PRJNA843886^1^ after the indicated release date.

### Global metabolome analysis

Feces were collected on days 28 following operation for metabolite analysis. The metabolites were isolated using conventional liquid–liquid extraction procedures as previously described (Jang et al., 2021). In brief, 3–4 volumes of chloroform/methanol (2/1, v/v) were added to freeze-dried feces samples and then centrifuged for 15 min. Polar metabolites were collected in the upper aqueous phase and nonpolar metabolites containing lipids were collected in the lower organic phase. Liquid chromatography/Mass Spectrometry (LC/MS) analysis was performed for each sample solution in both positive and negative ion modes. Compounds were identified by comparison to library entries of purified standards or recurrent unknown entities. Metabolites showing statistically significant changes (*p* < 0.05) were chosen and identified according to the KEGG database. Statistical analyses were conducted using MetaboAnalyst 4.0.

### Statistical analysis

To determine differences between groups at a single time point, normally distributed parametric data were tested using a two-way Student’s t test (for two groups) or one-way ANOVA followed by Tukey’s multiple comparisons test (for more than two groups). Nonparametric data were tested using Mann–Whitney’s U test. The Kaplan–Meier method and Log-rank (MantelCox) Test were used to construct and compare the survival curves of animals, respectively. All analyses were performed using Prism 6 software (GraphPad), and only differences with a *p* value less than 0.05 were considered statistically significant.

## Results

### High fiber diet preserves cardiac function, limits infarct size, and inhibits adverse remodeling following myocardial infarction

**Figure 1:**
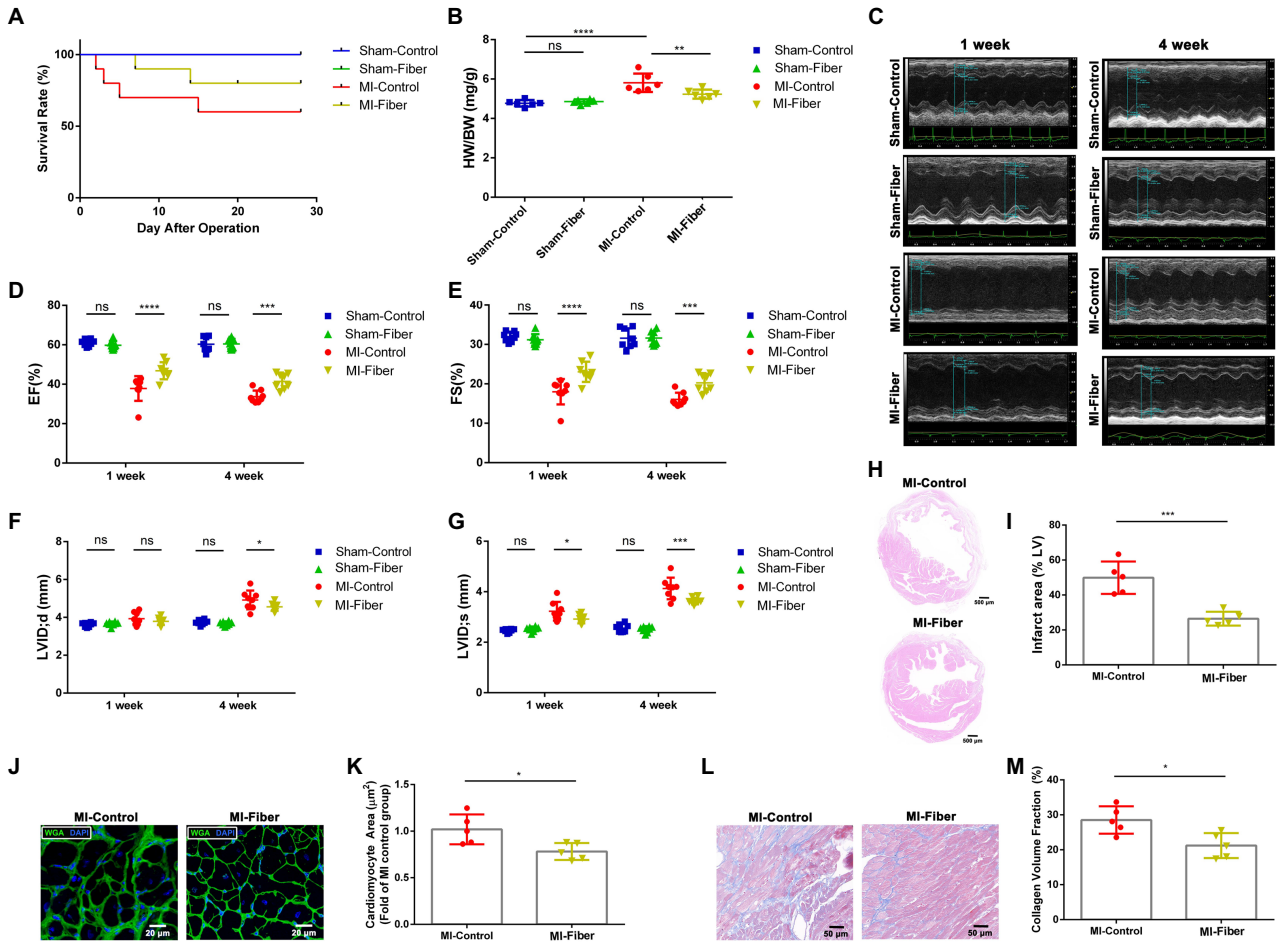
High fiber diet alleviates myocardial infarction and left ventricular remodeling in mice. **(A)** Survival rate of control diet or high fiber diet treated mice after MI. **(B)** Heart/body weight ratios of sham-control, sham-fiber, MI control and MI fiber mice 4 weeks post operation (*n* = 6). **(C)** Representative echocardiography M-mode images of control diet or high fiber diet treated animals 1 and 4 week following operation. **(D**,**E)** Quantitative analysis of EF% **(D)** and FS% **(E)** measured by echocardiography 1 and 4 week following MI (*n* = 8). **(F**,**G)** Quantitative analysis of LVIDd **(F)** and LVIDs **(G)** measured by echocardiography 1 and 4 week following MI (*n* = 8). **(H)** Representative images of HE stained heart tissues 4 weeks following MI. Scale bar = 500 μM. **(I)** Quantification of infarct area (%) within the ischemic heart in **(H)** (*n* = 5). **(J)** Representative images of WGA staining of peri-infarct zone 4 weeks post MI. Scale bar = 20 μM. **(K)** Quantitative analysis of cardiomyocyte cross-sectional area in **(J)** (*n* = 5). **(L)** Representative images of Masson’s trichrome stained peri-infarct zone from control diet or high fiber diet mice 4 weeks following MI. Scale bar = 50 μM. **(M)** Quantification of interstitial fibrosis by calculating collagen volume fraction in **(L)** (*n* = 5). Graphs depict mean ± SD. *p < 0.05, **p < 0.01, ***p < 0.001, ****p < 0.0001, ns, not significant.

To explore the role of high fiber diet in MI, we administered high fiber diet or control diet to male mice 3 weeks prior to induction of MI or sham operation. Survival rate was subsequently monitored over the period of a 28-day follow-up after surgery. When administering the high fiber diet, we found that the mortality rate of mice was apparently decreased compared to control diet group (Figure 1A). After sacrifice, we found that MI induction led to significant increase of heart weight/body weight ratio, while mice treated with high fiber diet showed near-normalized left ventricular (LV) to body weight ratio (Figure 1B). The heart tissues from the MI fiber group exhibited less spherical shape than those from the MI control mice at 4 weeks post operation in gross morphology (Supplementary Figure S1A).

To verify whether high fiber diet affects the cardiac function after MI, we performed echocardiography at 1 and 4 weeks post-operation. After induction of MI, all mice exhibited significant functional impairment at 1 and 4 week in comparison to the sham-treated mice. The mice of high fiber group showed a significant improvement in left ventricle ejection fraction (EF%) and fractional shortening (FS%) at 1 and 4 week compared with that of control diet group (Figures 1C–E). Moreover, MI induced dilation of LV end-systolic diameter (LVID;d) and LV end-diastolic diameter (LVID;s) was obviously blunted by high fiber treatment, indicating attenuation of adverse remodeling by high fiber diet (Figures 1F,G). Moreover, H&E staining revealed a significant reduction in the infarct size of mice treated with high fiber diet (Figures 1H,I).

Ventricular remodeling following MI is characterized by cardiomyocyte necrosis, cardiac fibrosis and compensatory myocardial hypertrophy. WGA staining was performed to measure the size of cardiomyocytes close to the infarct zone. As shown in Figures 1J,K, administration of high fiber diet significantly reduced hypertrophy of cardiomyocytes relative to control diet. We also performed Masson staining to evaluate the extent of cardiac fibrosis. The results revealed a significant decrease of interstitial collagen deposition in hearts of high fiber diet treated mice compared with those of the control diet group, suggesting the anti-fibrosis effect of dietary fibers (Figures 1L,M).

### High fiber diet modulates monocyte infiltration and attenuates inflammation following myocardial infarction

**Figure 2:**
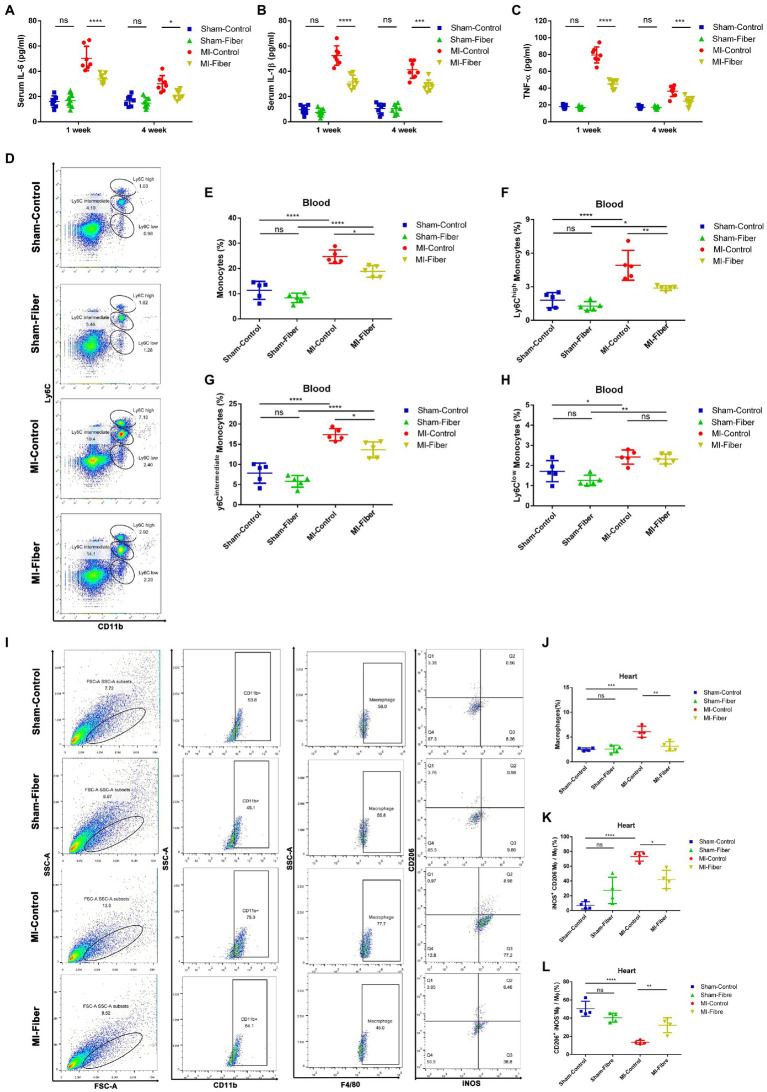
High fiber diet attenuates inflammation, prevents monocytes infiltration and promotes macrophage polarization following MI in mice. **(A–C)** Concentration of cytokines IL-6 **(A)**, IL-1β **(B)**, and TNF-α **(C)** in serum 1 and 4 week following MI. **(D)** Representative flow cytometry plots showing the gating strategy used to determine total monocytes (CD11b^+^Ly6C^+^), Ly6C^high^ monocytes (CD11b^+^Ly6C^high^), Ly6C^int^ monocytes (CD11b^+^ Ly6C^intermediate^) and Ly6C^low^ monocytes (CD11b^+^ Ly6C^low^) in peripheral blood 7 days following operation. **(E–H)** Quantification of total monocytes **(E)**, Ly6C^high^ monocytes **(F)**, Ly6C^int^ monocytes **(G)** and Ly6C^low^ monocytes **(H)** in peripheral blood of control diet or high fiber diet treated mice 7 days following operation (*n* = 5). **(I)** Representative flow cytometry plots for cardiac tissue in control diet or high fiber diet treated mice 7 days post operation. Live cells were first gated with CD11b and F4/80 positive (CD11b^+^F4/80^+^) population to identify macrophages. Macrophage were then gated into iNOS^+^CD206^−^ population and iNOS^−^CD206^+^ population, which represent M1 phenotype and M2 phenotype, respectively. **(J–L)** Pooled flow cytometry data from I (*n* = 4). Graphs depict mean ± SD. *p < 0.05, **p < 0.01, ***p < 0.001, ****p < 0.0001, ns, not significant.

Due to the well-established role of inflammation in the development of MI, we tried to elucidate the effect of high fiber diet on inflammatory cascade following MI. We examined the levels of pro-inflammatory cytokines in mice serum and found that either 1 or 4 week following MI, the levels of IL-1β, IL-6, and TNF-α were all elevated in serum of MI mice as compared with the sham-operated mice. The increases of IL-6, IL-1β, and TNF-α in serum of high fiber diet treated mice were apparently milder versus the control diet treated mice (Figures 2A–C).

Since previous findings and our studies declared that macrophages were critical in the modulation of cardiac inflammation following MI (Ong et al., 2018; Zhao et al., 2019), we performed flow cytometry analysis to assess the quantity and subpopulation of monocytes in peripheral circulation and macrophages in ischemic myocardium of high fiber diet treated and control diet treated mice 1 week following MI. Flow cytometric analysis demonstrated a significant reduction of monocytes, especially pro-inflammatory Ly6C^high^ monocytes, in peripheral blood of high fiber diet treated MI mice compared with that of control diet treated MI mice (Figures 2D–H). In heart tissues, we found that the proportion of CD11b^+^F4/80^+^ macrophages was significantly increased in control diet treated MI mice as compared with the sham-operated mice. The ratio of inflammatory M1 macrophages (iNOS^+^CD206^−^) was remarkably increased, whereas the ratio of reparative M2 macrophages (iNOS^−^CD206^+^) was significantly decreased in control diet treated group when compared with sham-operated group. High fiber diet significantly reduced the CD11b^+^F4/80^+^ macrophage population in cardiac tissue. Furthermore, compared with control diet treated mice, the increase of M1 macrophages and decrease of M2 macrophages were partially reversed in mice treated with high fiber diet, indicating that high fiber diet attenuated cardiac inflammation through regulating the balance between M1 and M2 macrophages (Figures 2I–L).

### High fiber diet modulates the gut microbial community structure

**Figure 3:**
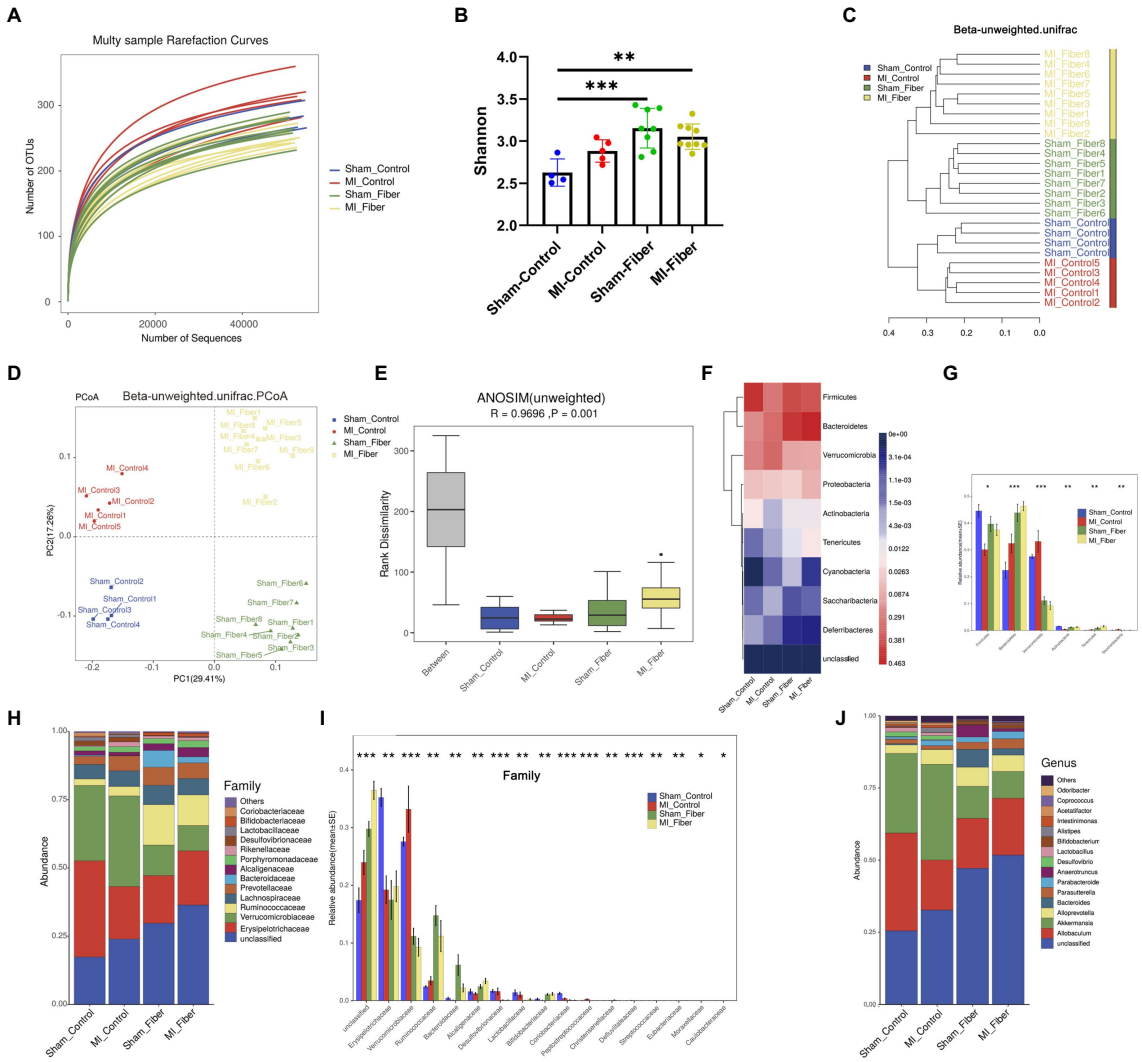
High fiber diet alters the composition of the gut microbial community. **(A)** Rarefaction curves of each sample. **(B)** Bacterial α-diversity estimated by Shannon’s diversity among groups. **(C)** Samples’ clustering result (Description, Beta unweighted_unifrac). **(D)** Bacterial β-diversity of principal coordinates analysis (PCoA) based on unweighted_unifrac distance indicated a distinctive distribution of the colonic microbial communities among all groups. **(E)** Anosim similarity analysis based on unweighted_unifrac distance rarefaction curves of each sample. **(F)** Heat map depicting gut microbial composition at phylum level. **(G)** Relative abundance of significantly different taxa at phylum level. **(H**,**I)** Proportion and relative abundance of different taxa at family level. **(J)** Proportion of different taxa at genus level. Sham control, *n* = 4; Sham fiber, *n* = 8; MI control, *n* = 5; MI fiber, *n* = 9. *p < 0.05, **p < 0.01, ***p < 0.001, ****p < 0.0001.

Accumulating evidence declared that dietary fibers have a major impact on intestinal flora composition, diversity and richness (Marques et al., 2017; Cronin et al., 2021). The morphology of whole intestine tract also changed apparently under high fiber diet relative to control diet (Supplementary Figure S1B). We further investigated whether high fiber supplementation affected the intestinal flora composition in sham and MI mice by 16S rRNA gene sequencing. Rarefaction analysis suggested that the estimated OTU richness could approach saturation in each sample (Figure 3A). The OTU counts index in MI Fiber group were significantly lower than that of MI control group. Likewise, compared with MI control mice, the α diversity of gut microbiota in MI Fiber mice was significantly decreased as determined by Chao index, ACE index and phylogenetic diversity (Figure 3B; Supplementary Figure S2). β diversity, evaluated by the unweighted unifrac cluster tree was different in both fiber diet groups in comparison to the control diet groups (Figure 3C). We then visualized unweighted UniFrac dissimilarity by principal coordinate analysis (PCoA), which suggested different overall gut microbial community structures among the four groups (Figure 3D). Additionally, Anosim analysis based on unweighted unifrac distance also disclosed a distinctive intestinal flora composition in MI Fiber and sham Fiber groups when compared with the two control diet groups (Figure 3E; Supplementary Figure S3).

Furthermore, the differences in the gut microbiota composition were evaluated based on the phylum, family, genus and species levels (Figures 3F–J; Supplementary Figures S4, S5). At phylum level, significant changes were found in Firmicutes, Bacteroidetes, Verrucomicrobia, Actinobacteria, Tenericutes, and Saccharibacteria phylum among four groups, suggesting the great impact of high dietary intake on gut microbial community (Figures 3F,G). Consistent with changes in phylum level, dramatic differences were observed among four groups at the family level, especially in Verrucomicrobiaceae, Lactobacillaceae, Bacteroidaceae and Bifidobacteriaceae family (Figures 3H–J and Supplementary Figure S4). We further analyzed the subordination of these different bacterial strains by linear discriminant analysis effect size (LEfSe) analysis and found that high fiber diet resulted in an apparent reduction of multiple bacterial species, including Akkermansia_muciniphila from Akkermansia genus and Verrucomicrobiaceae family, Lactobacillus_murinus from Lactobacillus genus and Lactobacillaceae family. Moreover, high fiber diet caused a significant increase of Bacteroides_acidifaciens from Bacteroides genus and Bacteroidaceae family, Bifidobacterium_choerinum from Bifidobacterium genus and Bifidobacteriaceae family (Supplementary Figures S4, S5A–D). Additionally, distinct differences in the functional potential of gut microbiomes predicted by PICRUSt were detected between MI control and sham control group, while minor differences were found between MI fiber and sham fiber group, indicating that high fiber diet apparently restored the normal function of gut microbiota (Supplementary Figures S5E–I). Together, these data suggested that high fiber supplementation modulates the gut microbial community structures and effectively alters the composition of gut microbiota following MI. These findings motivated us to investigate whether the beneficial effects of a high-fiber diet can cause a shift in microbial composition thereby inducing changes in gut-microbiota-derived metabolites.

### High fiber diet modulates the global metabolomic profile

**Figure 4:**
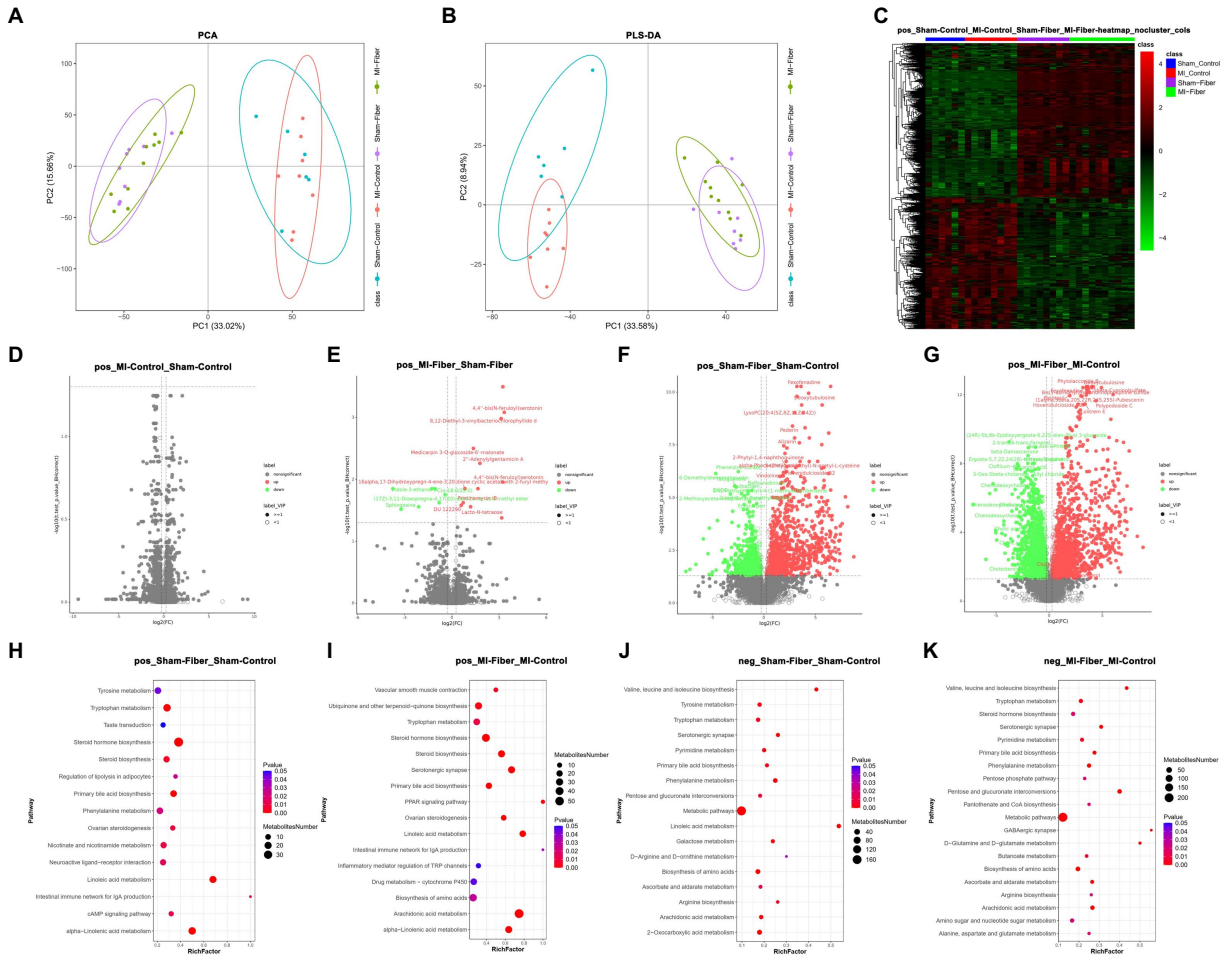
High fiber diet alters gut derived metabolites. **(A**,**B)** Principal component analysis analysis **(A)** and partial least squares discriminant analysis analysis **(B)** showed a distinctive metabolites composition among four groups. **(C)** Heat map depicting global metabolomic profiles among four groups. **(D–G)** Volcano plot disclosing the differences in metabolites between two groups in following ways: sham control vs. MI control **(D)**, sham fiber vs. MI fiber **(E)**, sham fiber vs. sham control **(F)** and MI control vs. MI fiber **(G)**. **(H–K)** Bubble chart distributing significantly different associated metabolic pathway between fiber diet groups and control diet groups in positive ions mode **(H**,**I)** and negative ions mode **(J**,**K)**. Sham control, *n* = 6; Sham fiber, *n* = 8; MI control, *n* = 8; MI fiber, *n* = 10.

Next, we investigated the impact of dietary fiber on gut derived metabolites using mass spectrometry. Fecal samples from each group were retrieved for metabolite analysis using MetaboAnalyst 4.0. We first visualized dissimilarity by principal component analysis (PCA) and partial least squares discriminant analysis (PLS-DA), which declared different metabolomic profile among sham control, MI control, sham fiber and MI fiber group. We found the samples of control diet groups clustered together and were far away from the fusion clustering of high fiber diet groups (Figures 4A,B), indicating the apparent influence of high fiber diet on intestinal metabolites. Additionally, heat map and volcano plot also disclosed a distinctive metabolites composition in sham fiber and MI fiber groups when compared with the corresponding control diet group, while the impact of MI induction on metabolites was relatively small (Figures 4C–G).

We then performed pathway analysis using a KEGG library to identify significantly changed metabolic features. The comparisons between two groups were further analyzed in following ways: (1) sham control vs. MI control, (2) sham fiber vs. MI fiber, (3) sham fiber vs. sham control and (4) MI control vs. MI fiber (Figures 4H–K and Supplementary Figure S6). Several common pathways were significantly affected by high fiber diet. Linoleic acid metabolism, primary bile acid synthesis, amino acids biosynthesis and steroid hormone biosynthesis exhibit a significant effect. Among these pathways, linoleic acid metabolism showed high rich factor and low *p* value (<0.01), primary bile acid biosynthesis had significant impact in both comparisons. Previous studies demonstrated that lower plasma level of linoleic acid was associated with increase in cardiovascular risk (Pertiwi et al., 2020; Nielsen et al., 2021; Nilsen et al., 2021). Increased levels of these linoleic acid metabolism related metabolites induced by high fiber diet might be involved in the protective effect of dietary fiber in MI. On the other hand, primary bile acid metabolic products from commensal microbiota was reported to play a role in inflammation modulation in cardiovascular diseases (Joyce and Gahan, 2017). The protective effects of high fiber diet against MI progression might be influenced by the interactions of gut microbiota, metabolites and immune system.

## Discussion

In this study, we firstly demonstrated that high fiber diet could improve cardiac function and prevent adverse remodeling following MI. The curative effects of high fiber diet in MI have a strong relation with its modulation of inflammation, especially macrophages. Then we explored whether high dietary fiber intake affected the composition of gut microbiota and played a role in the production of metabolites. The changes of gut microbiota and microbiota derived metabolites suggested that the protection exhibited by high fiber diet against MI might depend on the gut microbiota-metabolites-immunity interactions.

Western lifestyle, particularly excessive intake of sodium and fat, has been consistently associated with the incidence of hypertension and cardiovascular diseases (De Pergola and D’Alessandro, 2018; Kaye et al., 2020; Van Hecke et al., 2021). An inverse relationship between dietary fiber intake and the risk of cardiovascular diseases is well established (Wang et al., 2014; Wu et al., 2015; Soliman, 2019). Adequate fiber intake has been considered an important element for the prevention of cardiovascular diseases. However, the influence of high fiber diet on cardiac repair after MI has been rarely studied. In our study, we used a MI mouse model to investigate the therapeutic potential of high fiber diet in MI. We found that supplementation with dietary fiber led to remarkable protective effects on MI, manifested as reduced mortality, preserved cardiac function, smaller infarct size and attenuated adverse remodeling. Our findings suggested that dietary fiber not only prevented the incidence of cardiovascular disease, but also exerted therapeutic effects on MI.

Cardiac inflammation plays an important role in the development of MI by exacerbating tissue damage and accelerating the cardiac fibrotic response (Prabhu and Frangogiannis, 2016). Recent interest has emerged in regard to the ability of dietary fiber to modulate the intensity of inflammation in asthma and inflammatory bowel disease (Trompette et al., 2014; Jadhav et al., 2020), indicating the immunomodulatory potential of dietary fiber. In our work, we found that the MI-fiber group seems to show a reduced recovery at 4 weeks compared to the first week. Following MI, the intensity of inflammation reached the peak within the first week following myocardial infarction and gradually transforming to the anti-inflammatory reparative phase, which allows wound healing, and scar formation. So we further investigated whether the curative effects of high fiber diet had a strong relation with its modulation of cardiac inflammation. We demonstrated that inflammatory cytokine levels were lower in MI mice treated with high fiber diet relative to control diet group, suggesting the ant-inflammatory effect of high fiber diet. Recent studies have highlighted the importance of monocytes/macrophages in cardiac inflammation (Ong et al., 2018; Kologrivova et al., 2021). Macrophages contributed to both the initiation and resolution of inflammation, which is necessary for cardiac repair. After MI, there is an early influx of inflammatory monocytes in the initial inflammatory process, which gives rise to classically activated inflammatory (M1) macrophages and create a pro-inflammatory environment for necrotic debris clearance, and gradually replaced by reparative (M2) macrophages in the latter reparative process (Ben-Mordechai et al., 2013; van der Laan et al., 2014). Our study demonstrated that the numbers of monocytes in peripheral circulation, especially pro-inflammatory Ly6C^high^ monocytes, were greatly reduced in the high fiber diet group following MI. Furthermore, we declared that high fiber diet not only attenuated the infiltration of macrophages in ischemic myocardium but also boosted the transition of pro-inflammatory macrophages toward reparative macrophages.

Recently, growing evidence indicated that the intestinal flora is crucial in maintaining host homeostasis *via* immune system modulation (Rooks and Garrett, 2016). The interactions between dietary fiber and intestinal flora communities were investigated under the circumstance of MI. Our study showed that high fiber supplementation modulated the intestinal flora community structures and effectively altered the composition of the intestinal flora. Consistent with previous study (Marques et al., 2017), the abundance of Bacteroidaceae family members, major producers of short chain fatty acids (SCFAs), were significantly increased in high fiber diet groups. There is strong evidence that SCFA receptor signaling may influence the migration of immune cells and suppress inflammatory cytokines production (Kaye et al., 2020; Pakhomov and Baugh, 2021). The increase of SCFAs producing bacteria induced by high fiber diet indicated a strong link between dietary fiber, gut microbiota and the inflammation attenuation in the development of MI. Further analysis showed that high fiber diet significantly increased the abundance of the bacteria Bacteroides_acidifaciens. This bacteria has been shown to improve insulin sensitivity and prevent obesity in mice (Yang et al., 2017), but its role in the development of MI needs further investigation. In brief, our findings provide evidence for the importance of heart-gut axis in the pathogenesis of MI.

Several studies have highlighted the importance of metabolites produced by the gut microbiota in host immunity (Rooks and Garrett, 2016; Vernocchi et al., 2020). Global metabolomics profile analysis was performed in the current study. As expected, numerous metabolites and relevant metabolic pathways were affected by high fiber diet. Notably, linoleic acid metabolism pathway represented the most significant change in metabolic pathways. Previous reports suggested a cardioprotective effect of high intakes of linoleic acid (Smit et al., 2010). Moreover, metabolic products of linoleic acid metabolism pathway were found to have anti-inflammatory, anti-proliferative and antithrombotic properties (Jang et al., 2021). Apart from linoleic acid metabolism, high fiber diet also affected bile acid biosynthesis. These microbiota generated bile acids are particularly potent signaling molecules that interact with host bile acid receptors (such as farnesoid X receptor, vitamin D receptor and G protein-coupled BA receptor 1) to trigger cellular responses that play critical roles in electrolyte transport, host lipid metabolism and immune modulation (Ridlon et al., 2016). These results suggested that the curative effect exerted by high fiber diet against MI might depend on the microbiota-metabolism-immunity interactions. However, the complex relationship and underlying mechanisms among metabolites-immunity interactions in MI needs further investigation.

## Conclusion

In summary, the findings of our study indicated that compositions of intestinal flora and intestinal metabolites could be modulated by a high fiber diet, consequently preventing MI progression *via* attenuation of local and systemic inflammation, which might provide potential therapeutic strategy for MI treatment.

## Data availability statement

The data presented in the study are deposited in the NCBI repository, accession number PRJNA843886.

## Ethics statement

The animal study was reviewed and approved by the Institutional Ethics Committee of Nanjing Drum Tower Hospital (Approval No. 20011141).

## Author contributions

JZ, WC, LK, JX, and BX: conceptualization and writing—review and editing. JZ, WC, HL, and AS: methodology and investigation. WC, HL, and XS: formal analysis. JZ and WC: data curation. JZ: writing—original draft preparation. QZ and AS: visualization. LK, JX, and BX: project administration. XS: supervision. JZ, WC, and BX: funding acquisition. All authors have read and agreed to the published version of the manuscript.

## Funding

This work was supported by the National Natural Science Foundation of China (grant numbers 82000264, 82070366, and 82100533); Natural Science Foundation of Jiangsu Province (grant number BK20200139); the Funds for Distinguished Young Scientists in Nanjing (JQX22001) and Jiangsu “Mass Innovation and Entrepreneurship” Talent Program to JZ.

## Conflict of interest

The authors declare that the research was conducted in the absence of any commercial or financial relationships that could be construed as a potential conflict of interest.

## Publisher’s note

All claims expressed in this article are solely those of the authors and do not necessarily represent those of their affiliated organizations, or those of the publisher, the editors and the reviewers. Any product that may be evaluated in this article, or claim that may be made by its manufacturer, is not guaranteed or endorsed by the publisher.

## Supplementary material

The Supplementary material for this article can be found online at: https://www.frontiersin.org/articles/10.3389/fmicb.2022.1046912/full#supplementary-material

## Footnotes

^1^ www.ncbi.nlm.nih.gov/sra/PRJNA843886
