# Supplementary material for: High fiber diet attenuate the inflammation and adverse remodeling of myocardial infarction via modulation of gut microbiota and metabolites
Source: Front Microbiol. 2022 Dec 21;13:1046912. doi: 10.3389/fmicb.2022.1046912 (PMC9810810; doi:10.3389/fmicb.2022.1046912)
Supplement: Supplementary file 2 [file Data_Sheet_2.docx]

***Supplementary Material***


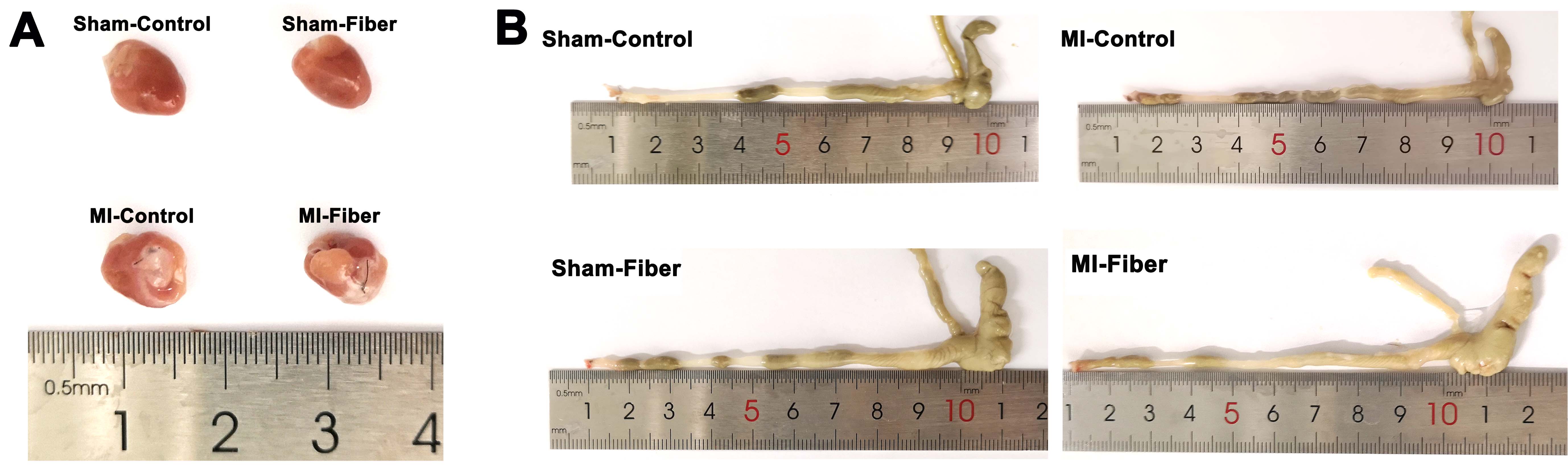


**Supplementary Figure 1.** Gross morphology of heart (A) and gastrointestinal tract (B) from control diet or high fibre diet treated mice 4 week following operation.


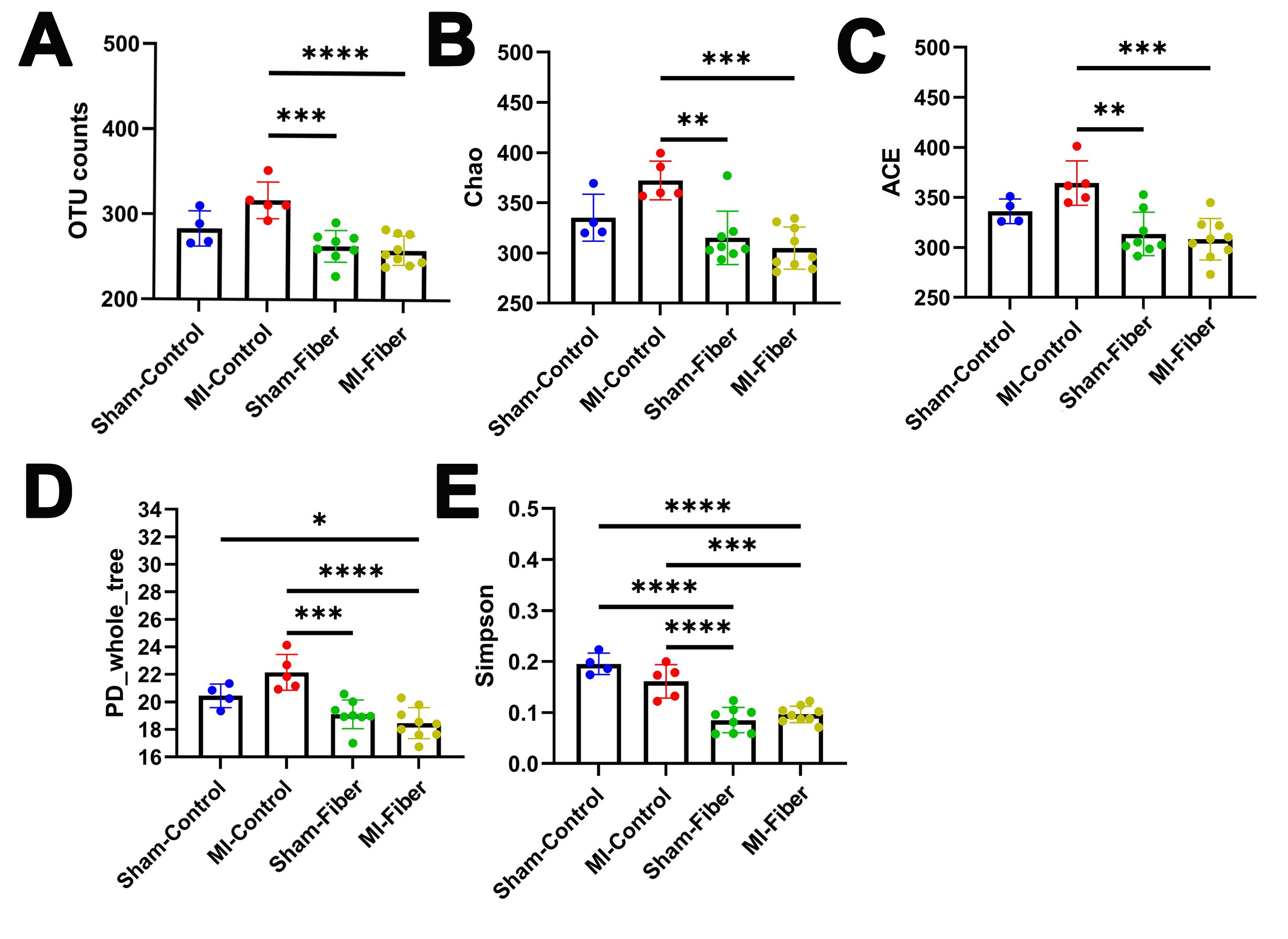


**Supplementary Figure 2.** Bacterial α-diversity estimated by OTU counts (A), Chao

(B), ACE (C), phylogenetic diversity (PD) (D) and Simpson’ diversity (E).


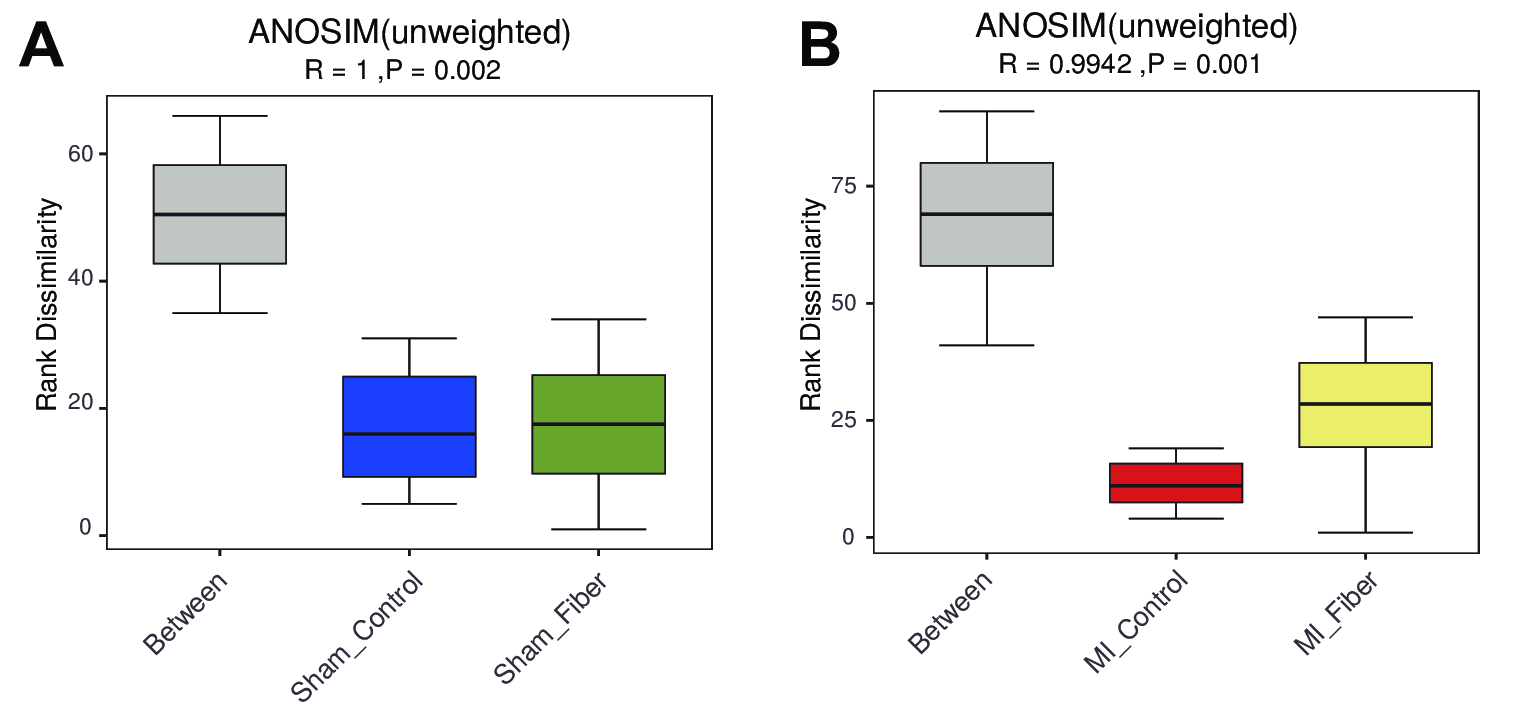


**Supplementary Figure 3.** Anosim similarity analysis based on unweighted_unifrac distance rarefaction curves between groups. (A) sham control vs. sham fibre (B) MI control vs. MI fibre.


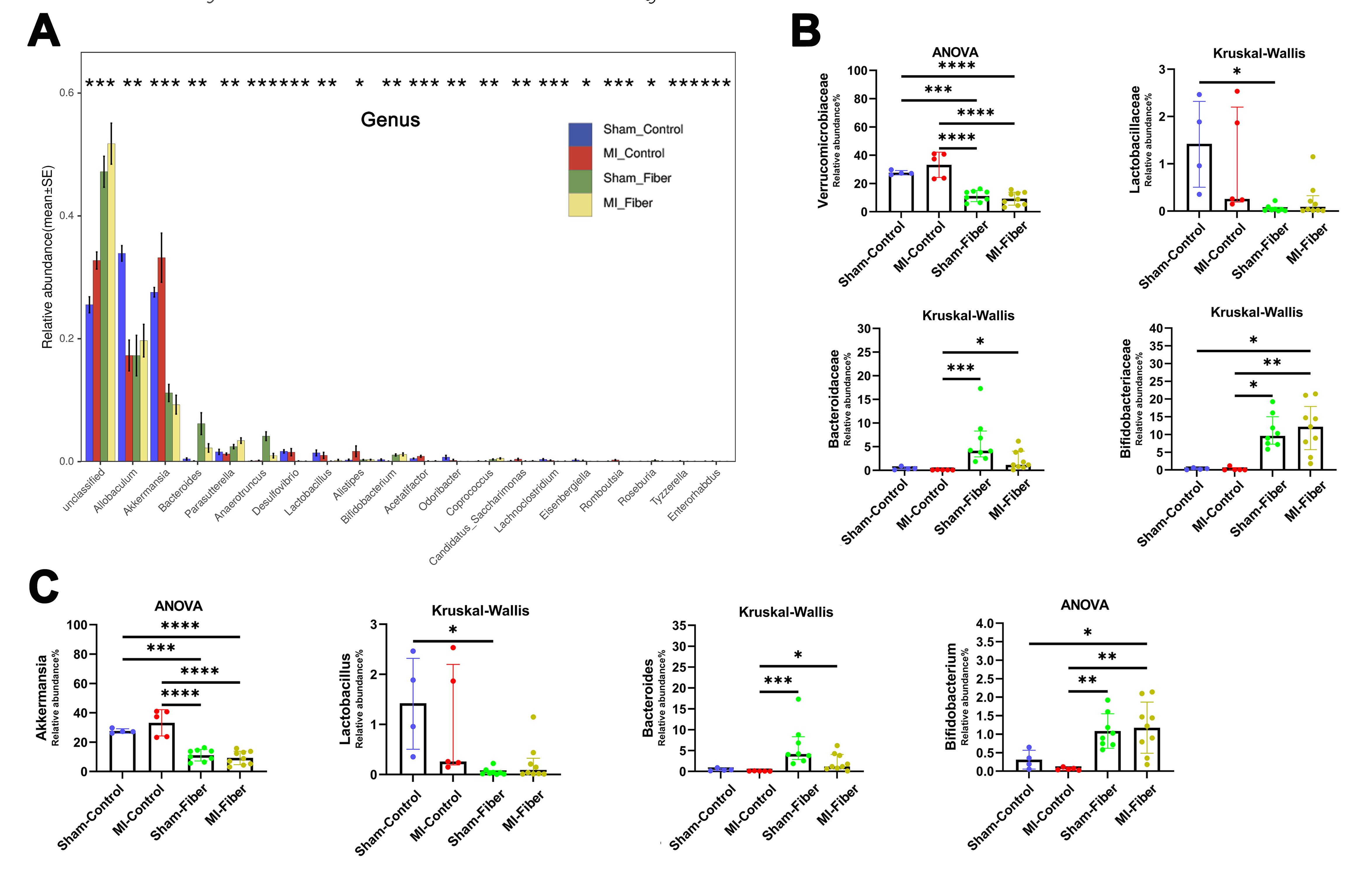


**Supplementary Figure 4.** High fiber diet ameliorates gut microbiota disorder. (A) Relative abundance of different taxa at genus level. (B) Relative abundance of Verrucomicrobiaceae, Lactobacillaceae, Bacteroidaceae and Bifidobacteriaceae among all groups. (C) Relative abundance of Akkermansia, Lactobacillus, Bacteroides and Bifidobacterium among all groups. Sham control, n=4; Sham fiber, n=8; MI control, n=5; MI fiber, n=9. *P < 0.05, **P < 0.01, ***P < 0.001, ****P < 0.0001.


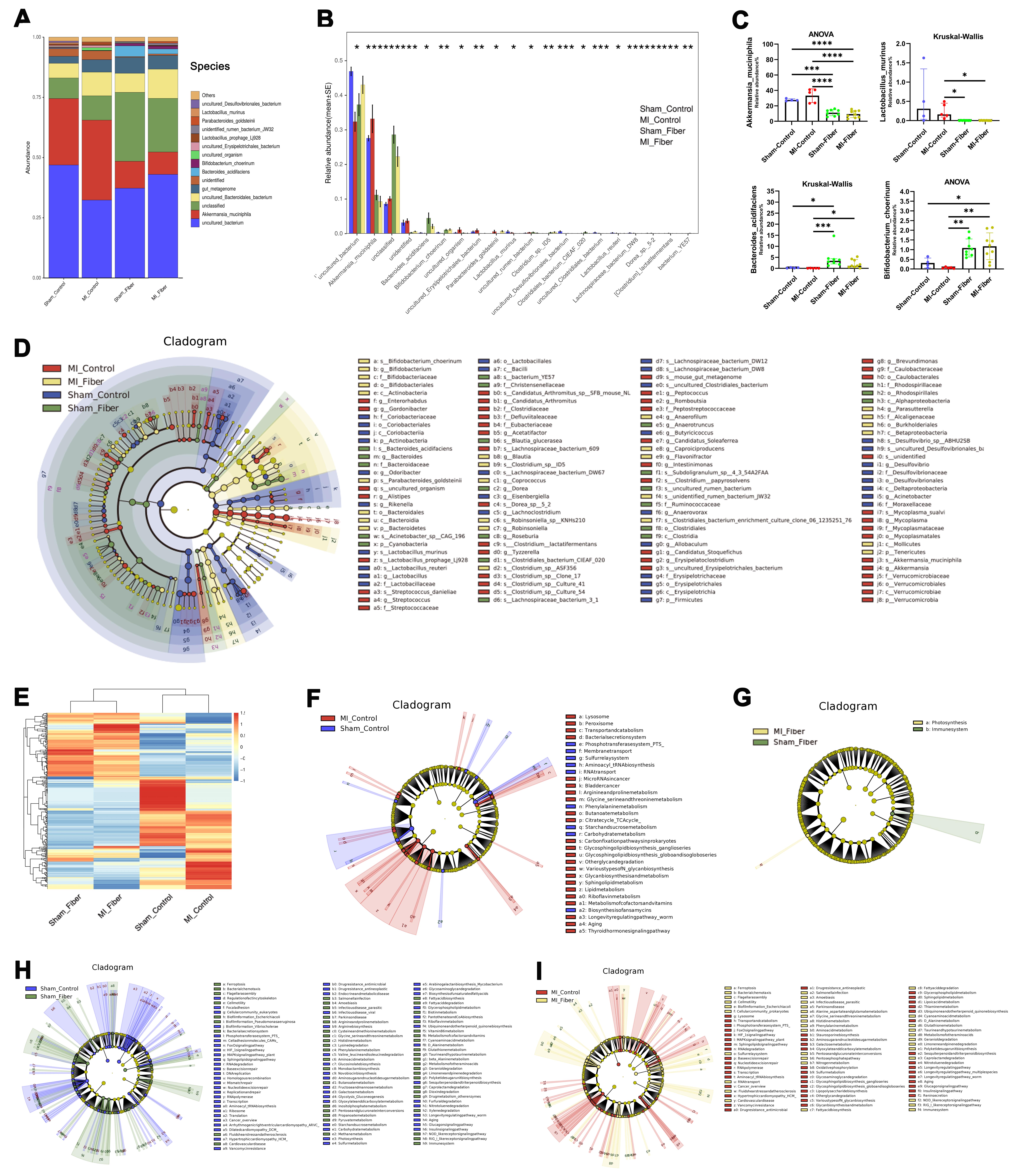


**Supplementary Figure 5.** The effect of high fibre supplementation in gut microbiome following MI. (A, B) Proportion and relative abundance of different taxa at species level. (C) Relative abundance of Akkermansia_muciniphila, Lactobacillus_murinus, Bacteroides_acidifaciens and Bifidobacterium_choerinum among all groups. (D) Cladogram showing the taxonomic hierarchical structure of the distinguished phylotype generated by linear discriminant analysis effect size (LEfSe) analysis. Each filled circle indicates a certain type of phylotype. Different colors represent different groups. Colored node consistent with the group color indicates important microbe biomarkers in the group and the name of biomarkers are listed in the right corner. (E) Heat map depicting predicted functional metagenomics pathways of gut microbiota that separated the four groups. (F-I) Cladogram identifying pathways that differentiated the sham control vs. MI control (F), sham fibre vs. MI fibre (G), sham fibre vs. sham control (H) and MI control vs. MI fibre (I). Each filled circle indicates a certain type of pathway. Different colors represent different groups. Colored node consistent with the group color indicates important functional pathways in the group and the name of pathways are listed in the right corner. Sham control, n=4; Sham fibre, n=8; MI control, n=5; MI fibre, n=9. *P < 0.05, **P < 0.01, ***P < 0.001, ****P < 0.0001.


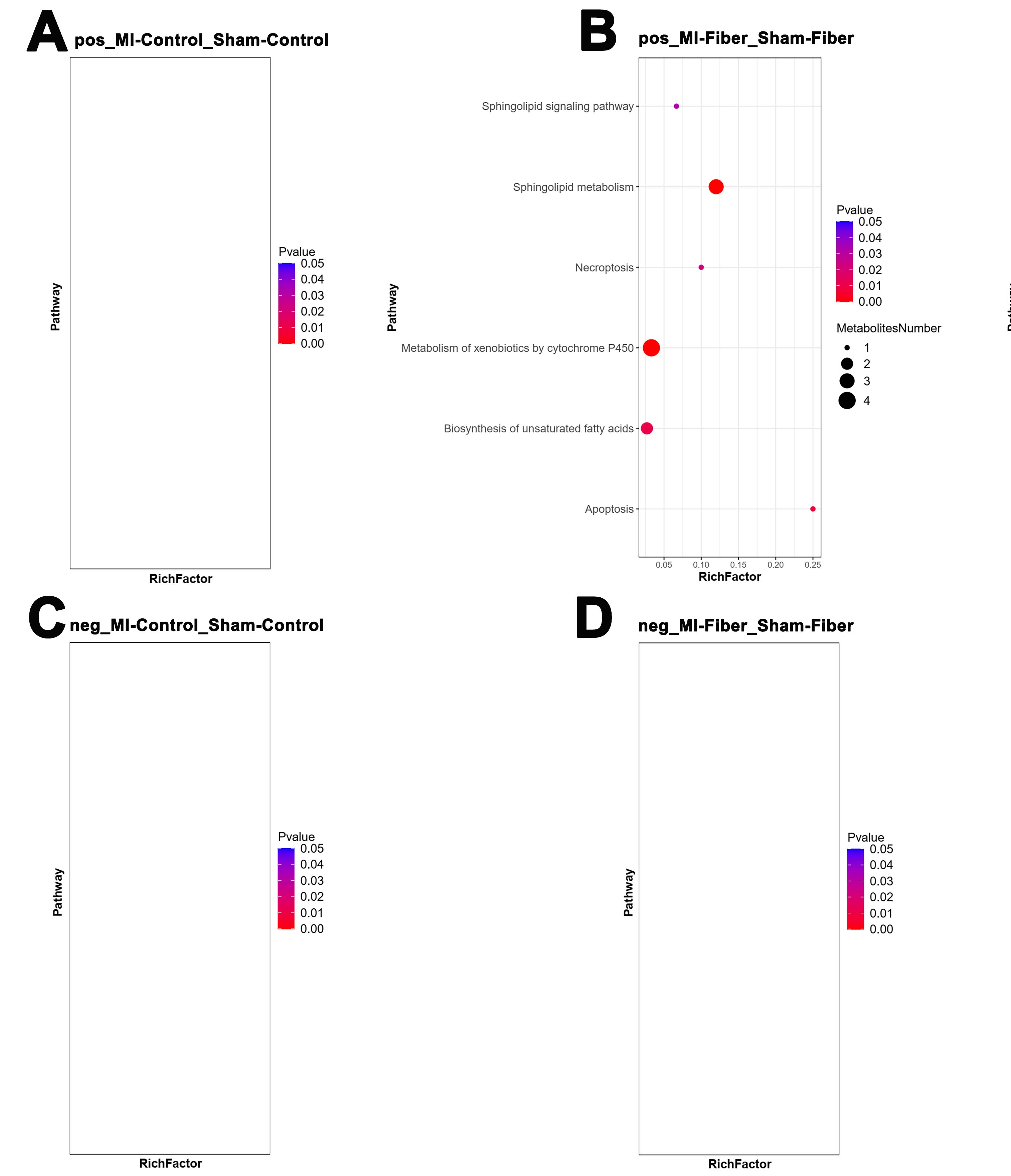


**Supplementary Figure 6.** Bubble chart distributing significantly different associated metabolic pathway between two groups in positive ions mode and negative ions mode. (A, C) sham control vs. MI control. (B, D) sham fibre vs. MI fibre.
